# Synaptophysin-like-1: A Novel Serum Diagnostic Marker for Pancreatic Ductal Adenocarcinoma Screening, Early Diagnosis, and Prognosis Prediction

**DOI:** 10.3390/jcm14113719

**Published:** 2025-05-26

**Authors:** Sefa Ergun, Taskin Avci, Seyma Dumur, Yasemin Pekmezci, Hafize Uzun, Uğurcan Sayılı, Yagmur Ozge Turac Kosem, Osman Simsek, Salih Pekmezci

**Affiliations:** 1 Department of General Surgery, Cerrahpaşa Faculty of Medicine, Istanbul University-Cerrahpasa, 34320 Istanbul, Turkey; sefaergn@yahoo.com (S.E.); taskinavci@gmail.com (T.A.); dryaseminpekmezci@gmail.com (Y.P.); yoturac@gmail.com (Y.O.T.K.); osman.simsek@iuc.edu.tr (O.S.); pekmezcisalih@gmail.com (S.P.); 2 Department of Medical Biochemistry, Faculty of Medicine, Istanbul Atlas University, 34403 Istanbul, Turkey; seyma_dumur@hotmail.com; 3 Department of Public Health, Cerrahpasa Faculty of Medicine, Istanbul University-Cerrahpasa, 34320 Istanbul, Turkey; ugurcan.sayili@iuc.edu.tr

**Keywords:** pancreatic ductal adenocarcinoma, metastatic pancreatic cancer, synaptophysin like 1 protein, CEA, CA19-9

## Abstract

**Background:** The role and underlying mechanisms of synaptophysin-like-1 (SYPL1), a neuroendocrine-associated protein, in pancreatic ductal adenocarcinoma (PDAC) remain unclear. This study aims to assess the diagnostic potential of SYPL1 as a serum biomarker for both resectable PDAC (rPDAC) and metastatic PDAC (mPDAC) located at the head of the pancreas. Additionally, the SYPL1 levels were monitored in PDAC patients who underwent surgical resection, with follow-up measurements taken 6 months postoperatively. **Method:** We analyzed serum SYPL1 in healthy controls (*n* = 67), rPDAC patients (*n* = 39), mPDAC patients (*n* = 22), and rPDAC patients (6-month postoperative) (*n* = 20) (due to factors such as relocation or death, 20 patients were included instead of 39 patients) by ELISA. **Results:** The SYPL-1 levels showed significant differences across the groups (controls: 7.43 ± 3.32, PC: 15.89 ± 2.00, mPDAC: 20.01 ± 4.03, *p* < 0.001). Both carcinoembryonic antigen (CEA) and carbohydrate antigen 19-9 (CA19-9) were significantly greater in cancer groups compared to the healthy group. In patients who underwent surgical resection, the SYPL-1 levels showed a significant decrease 6 months after surgery (*p* < 0.001). Strong correlations were observed between tumor markers, with CA19-9 showing a positive correlation with CEA in both rPDAC (r = 0.550, *p* < 0.001) and mPDAC (r = 0.623, *p* = 0.002), while SYPL-1 demonstrated a negative correlation with CEA (r = −0.530, *p* = 0.009) in mPDAC. Receiver operating characteristic (ROC) analysis revealed excellent diagnostic performance for SYPL-1 in distinguishing both rPDAC (AUC = 0.965) and mPDAC (AUC = 0.985) from healthy controls, achieving superior accuracy compared to conventional markers CEA and CA19-9. **Conclusions:** Serum SYPL-1 emerges as a promising biomarker for the diagnosis and monitoring of rPDAC and mPDAC. Its significantly elevated levels in cancer groups, coupled with its marked decrease following surgical resection, suggest that SYPL-1 could play a critical role in both initial diagnosis and post-treatment surveillance. The strong correlations observed between SYPL-1, CEA, and CA19-9 further support its potential utility in a multi-marker panel. Notably, SYPL-1 demonstrated superior diagnostic accuracy compared to conventional markers, with high AUC values indicating its excellent ability to distinguish rPDAC and mPDAC from healthy controls. These findings highlight the need for further investigation to validate SYPL-1 as a reliable, non-invasive biomarker that could enhance early detection, prognosis, and treatment monitoring in rPDAC.

## 1. Introduction

Pancreatic ductal adenocarcinoma (PDAC) is an aggressive malignancy characterized by a dense desmoplastic stroma with a poor prognosis. It is the seventh leading cause of cancer-related death worldwide. PDAC is asymptomatic in the early stages, and the characteristics of the tumor microenvironment, its ability to metastasize early, and its poor response to standard therapies by developing resistance to chemotherapeutic agents lead to low survival rates and recurrence [1,2]. However, the precise mechanisms underlying this disease remain unclear. Therefore, prevention, early detection and prompt treatment are clinically challenging [1].

Contrast-enhanced abdominal tomography, endoscopic retrograde cholangio pancreatography (ERCP), endoscopic ultrasonography (EUS) and biopsy can be used in the diagnosis of pancreatic cancer. Trans-abdominal biopsies should be preferred in irresectable cases due to the risk of peritoneal transplantation [3]. The guidelines mostly recommend triphasic abdominal computerized tomography (CT) for imaging [4]. Due to the lack of specific symptoms and markers, difficulty in anatomical localization and inadequate screening methods for early detection, pancreatic cancer is diagnosed only at late stages [3]. The only biomarker currently accepted by the Food and Drug Administration (FDA) and the National Comprehensive Cancer Network guidelines for pancreatic cancer is carbohydrate antigen 19-9 (CA19-9) [4,5]. Although CA19-9 can serve as a prognostic factor, its utility for early diagnosis and screening is limited [6,7]. Although CA 19-9 is the most frequently used biomarker in the clinic and the most researched biomarker, its sensitivity (70–92%) and specificity (68–92%) are not sufficient for early diagnosis and prognosis [8,9].

Synaptophysin-like 1 (pantophysin, SYPL1), a neuroendocrine-related protein, is abundant in adipocytes and is found in cellular membrane compartments overlapping with those containing the insulin-sensitive glucose transporter type 4 (GLUT4) [10]. SYLP1, which is also found in both neuronal and non-neuronal tissues, plays an important role in inflammation, tumor proliferation, and progression [11]. SYPL1 plays a vital role in colorectal cancer (CRC), PDAC, hepatocellular carcinoma (HCC), breast cancer (BC), and papillary thyroid carcinoma (PTC) [12,13,14,15,16,17].

There is currently no study in the literature that directly addresses our research objectives. Therefore, the aim of this study is to assess the diagnostic efficacy of SYPL1 as a serum biomarker for PDAC and metastatic PDAC (mPDAC) located at the head of pancreas. Additionally, we aimed to track changes in the SYPL1 levels in resectable PDAC (rPDAC) patients who underwent surgical resection, with follow-up measurements taken 6 months postoperatively.

## 2. Material and Methods

All subjects who participated in this study gave their informed consent and this study was approved by the ethics committee of Istanbul University-Cerrahpasa, Cerrahpas Medical Faculty (number: E-83045809-604.01.01-695875; date: 16 May 2023). This study was conducted in accordance with the Declaration of Helsinki.

Our study consists of patients who were admitted to the hepatopancreatobiliary (HPB) clinic of Istanbul University-Cerrahpaşa, Cerrahpaşa Medical Faculty, General Surgery, and diagnosed with pancreatic head ductal adenocancer between June 2023 and December 2024. This study included patients diagnosed with rPDAC based on pathology results in the general surgery outpatient clinic (*n* = 39), patients who underwent surgery for rPDAC and returned for follow-up at 6 months (*n* = 20) (due to factors such as relocation or death, 20 patients were included instead of 39 patients), and patients diagnosed with mPDAC (*n* = 22). The control group (*n* = 67) included healthy individuals. All the pancreatic malignancies were located at the head of the pancreas.

All patients with PDAC preoperatively underwent a dedicated contrast-material-enhanced multi-detector-row computed tomography with a pancreatic protocol, as previously described [18].

*Inclusion Criteria*

(i) Patients >18 years old; (ii) patients with newly diagnosed PDAC (with a prediagnosis of PDAC); (iii) underwent surgery.

*Exclusion Criteria*

(i) Having received chemotherapy or radiotherapy; (ii) pathological specimens could not be obtained; (iii) volunteers who did not sign the consent form; (iv) refused follow-up; (v) pancreatic adenocancer located outside the head of pancreas.

Venous blood samples were collected from all subjects in the morning, prior to breakfast. For patients, additional blood samples were collected 6 months after surgery. The blood samples were allowed to clot at room temperature for 30 min, followed by centrifugation at 1000× *g* for 20 min at 4 °C. The serum was carefully separated, aliquoted, and stored at −80 °C. Samples exhibiting hemolysis, icterus, or lipemia (HIL) were excluded from this study.

### 2.1. Serum Synaptophysin-like 1 (SYPL1) Analysis

Serum SYLP1 levels were measured using the Human SYLP1 ELISA Kit (MyBioSource, CA, USA). The coefficients of intra- and inter-assay variation were 5.6% (*n* = 15) and 7.4% (*n* = 15), respectively.

Tumor markers (CEA, CA19-9) were measured using an IMMULITE 2000 (DPC, Los Angeles, CA, USA).

Biochemical parameters were measured using the spectrophotometric method with an autoanalyzer (Hitachi Modular System, Roche Diagnostic, Corporation, Hague Road, Indianapolis, IN, USA). C-reactive protein (CRP) values were measured with the turbidimetric method with an auto analyzer (ADVIA 1800 Auto Analyzer, Siemens medical Sol., Deerfield, IL, USA).

### 2.2. Statistical Analyses

Statistical analyses were performed using SPSS 21.0 (IBM Corp., Armonk, NY, USA), JASP 0.18.3.0, and Jamovi 2.4.11. Categorical variables are presented as frequencies and percentages, while continuous variables are presented as mean ± SD or median and IQR. Normality was assessed with the Kolmogorov–Smirnov and Shapiro–Wilk tests. The chi-square test compared categorical variables, while continuous variables were compared using Kruskal–Wallis, one-way ANOVA, or the Mann–Whitney U test, depending on distribution. Post hoc analysis was conducted with Tukey’s test or adjusted *p*-values. Paired *t*-test or Wilcoxon test were used for dependent samples. ROC curve analysis evaluated the diagnostic performance of SYPL1, CEA, and CA19-9, with AUC, sensitivity, specificity, and optimal cut-off values calculated using the Youden index. A *p*-value < 0.05 was considered significant.

## 3. Results

The gender distribution showed significant differences among the study groups (*p* < 0.001). The proportion of females was 72.7% in the control group, 56.4% in the pancreatic cancer group, and 26.1% in the mPDAC.

The mean age was significantly different among groups (*p* < 0.001), with control subjects being younger (44.8 ± 13.84 years) compared to both rPDAC (64.57 ± 9.67 years) and metastatic pancreatic cancer patients (63.22 ± 9.44 years). The tumor diameter was similar between rPDAC [3.2 (2.8–4.5)] and mPDAC [4 (2.5–4.5)] (*p* = 0.645) (Table 1).

**Figure 1:**
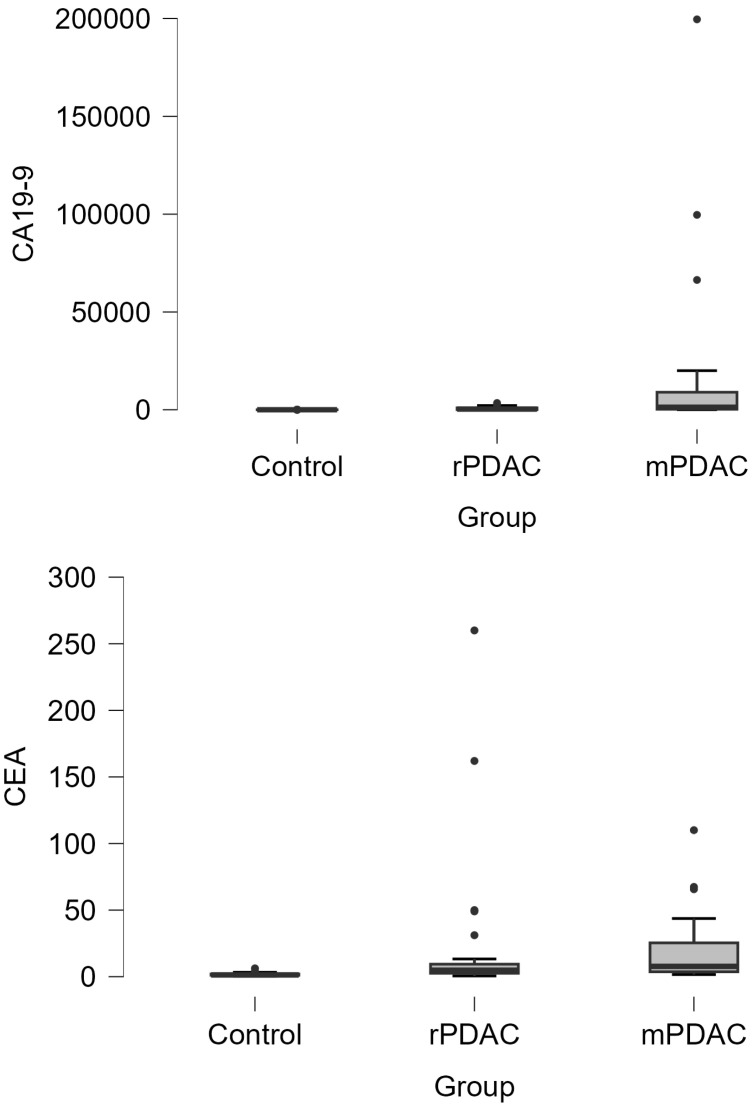

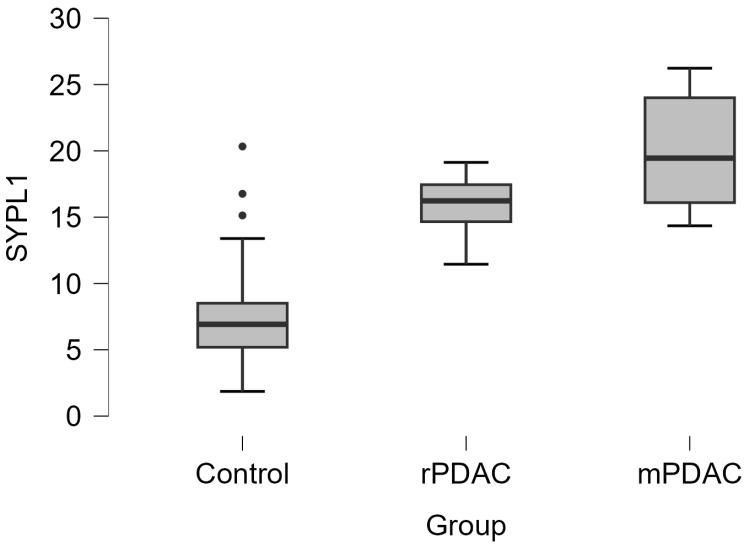
Box-plot distribution of SYPL-1, CEA, and CA19-9 levels across control, resectable pancreatic ductal adenocarcinoma (rPDAC), and metastatic (mPDAC) groups. Dots indicate extreme values.

The SYPL-1 levels showed a significant difference across the groups (*p* < 0.001), with mean values of 7.43 ± 3.32 in the controls, 15.89 ± 2.00 in rPDAC, and 20.01 ± 4.03 in mPDAC patients. The CEA levels were significantly elevated in cancer groups compared to the controls (*p* < 0.001), with median (IQR) values of 1.34 (1.06–2.00) in the controls, 4.86 (2.58–9.40) in rPDAC, and 7.74 (3.66–25.6) in mPDAC patients. Similarly, CA19-9 showed a marked elevation in cancer groups (*p* < 0.001), with median (IQR) values of 6.89 (4.42–10.6) in the controls, 330.25 (71.22–967.6) in rPDAC, and 1302.5 (251–9314) in mPDAC patients (Table 1, Figure 1).

Liver function tests showed significant differences across the groups. The AST levels were higher in cancer groups, with medians of 41 (16–104) in rPDAC and 25 (19–55) in mPDAC patients compared to 17 (15–24) in the controls (*p* < 0.001). The ALT levels were also elevated: 44 (14–136) in rPDAC and 28 (16–48) in mPDAC patients compared to 18.5 (11–27) in the controls (*p* = 0.001). Total bilirubin was higher in both cancer groups [pancreatic cancer: 1.3 (0.5–3.79), metastatic: 1.2 (0.43–2.84)] compared to the controls [0.37 (0.24–0.51)] (*p* < 0.001). The CRP levels increased progressively from the controls [2.31 (0.88–5.61)] to PDAC [6.5 (2.6–15.8)] and were highest in mPDAC patients [22.25 (8.5–57.8)] (*p* < 0.001) (Table 1).

The postoperative rPDAC group decreased from 39 to 20 patients due to factors such as relocation or mortality. In patients who underwent surgical resection, significant postoperative changes were observed. The SYPL-1 levels decreased from 15.82 ± 1.93 to 11.26 ± 2.22 (*p* < 0.001). The CA19-9 levels reduced from 330.13 (170–950.67) to 55 (44.55–78) (*p* < 0.001), while CEA remained stable (5.17 (2.4–9.59) to 7.7 (3.1–9.55), *p* = 0.173). Liver function improved, with ALP decreasing from 364 (115.5–550) to 102.5 (81–116) (*p* = 0.002) and GGT from 591.5 (109–949.5) to 40 (32–52) (*p* < 0.001). However, albumin levels decreased from 4.03 (3.5–4.32) to 3.22 (2.89–3.58) (*p* = 0.004) (Table 2).

**Figure 2:**
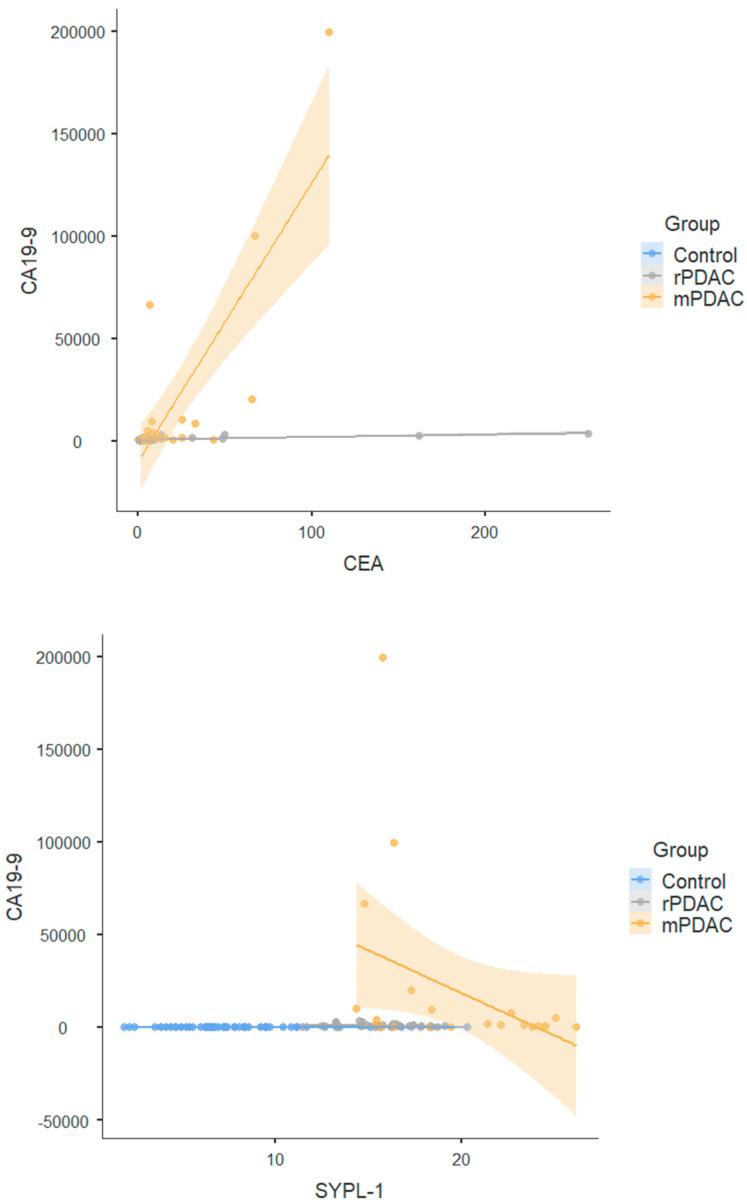

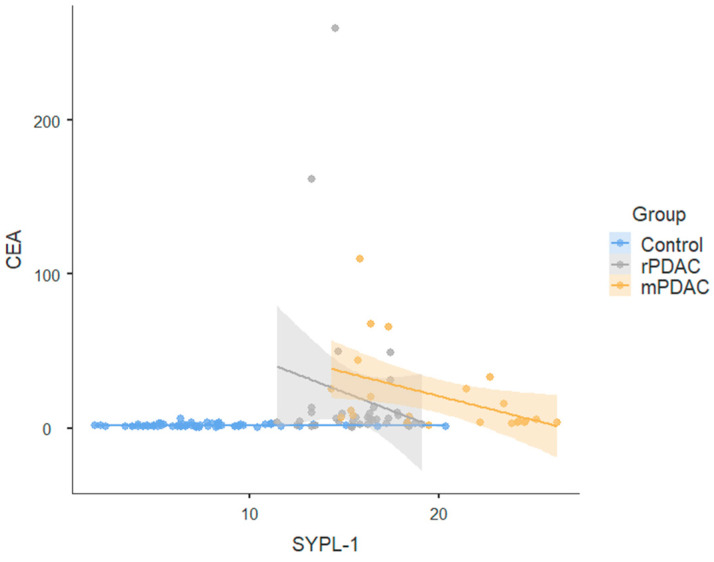
Correlation analysis of SYPL-1, CEA, and CA19-9 within control, resectable pancreatic ductal adenocarcinoma (rPDAC), and metastatic (mPDAC) groups.

In the rPDAC, CA19-9 demonstrated a strong positive correlation with CEA (r = 0.550, *p* < 0.001), while hematocrit (Hct) showed a moderate positive correlation with SYPL-1 (r = 0.329, *p* = 0.043) (Table 3, Figure 2).

In the mPDAC, CEA demonstrated a strong positive correlation with CA19-9 (r = 0.623, *p* = 0.002). Direct bilirubin levels positively correlated with tumor diameter (r = 0.424, *p* = 0.044) (Table 3, Figure 2).

ROC curve analysis assessed the diagnostic performance of SYPL-1, CEA, and CA19-9. SYPL-1 showed excellent accuracy with an AUC of 0.965 (95% CI: 0.928–1, *p* < 0.001), sensitivity of 92.4%, and specificity of 78.4% at a cut-off of 12. CEA had an AUC of 0.857 (95% CI: 0.773–0.941, *p* < 0.001), with 89.2% sensitivity and 88.1% specificity at a cut-off of 2.5. CA19-9 also performed excellently, with an AUC of 0.924 (95% CI: 0.857–0.991, *p* < 0.001), 82.1% sensitivity, and 100% specificity at a cut-off of 20. In addition, CA19-9 had 100% specificity at a cut-off of 60 (Table 4).

For distinguishing mPDAC from health, all markers showed enhanced performance. SYPL-1 had the highest accuracy with an AUC of 0.985 (95% CI: 0.964–1, *p* < 0.001), achieving 100% sensitivity and 92.4% specificity at a cut-off of 12, and 95.5% sensitivity and 100% specificity at a cut-off of 14. CEA showed excellent performance with an AUC of 0.965 (95% CI: 0.921–1, *p* < 0.001), with 95.7% sensitivity and 91.3% specificity at a cut-off of 2.5, and 91.0% sensitivity and 88.1% specificity at a cut-off of 3. CA19-9 performed outstandingly with an AUC of 0.986 (95% CI: 0.963–1, *p* < 0.001), achieving 95.5% sensitivity and 100% specificity at a cut-off of 20, and 90.9% sensitivity and 97.4% specificity at a cut-off of 60 (Table 4).

In differentiating mPDAC from rPDAC, SYPL-1 showed moderate diagnostic accuracy with an AUC of 0.771 (95% CI: 0.641–0.900, *p* = 0.001), achieving 52.2% sensitivity and 100% specificity at a cut-off value of 19, and 47.8% sensitivity and 97.3% specificity at a cut-off value of 20. CA19-9 demonstrated fair performance with an AUC of 0.701 (95% CI: 0.553–0.850, *p* = 0.010), with 40.9% sensitivity and 100% specificity at both cut-off values of 3000 and 3500. CEA showed limited diagnostic value in this distinction with an AUC of 0.647 (95% CI: 0.505–0.790, *p* = 0.060) (Table 4).

## 4. Discussion

Recent investigations into novel biomarkers have shown promise, but they require further validation with larger sample sizes and standardized measurement methods. Cost effectiveness and practicality are crucial considerations for the widespread clinical application of these biomarkers. The authors emphasize the importance of focused research on realistic and applicable early detection methods and models for clinical use [19]. PDAC is one of the most aggressive and challenging forms of cancer, often diagnosed at an advanced stage due to its asymptomatic nature in early phases. Early diagnosis is critical for improving patient outcomes, making the search for reliable serum biomarkers essential [20]. The most important finding of this study is that SYPL-1 levels were significantly elevated in both cancer groups, with the highest levels in mPDAC patients, compared to rPDAC and controls. CEA and CA19-9 were both significantly elevated in the cancer groups when compared to the control group. The inflammatory marker CRP was progressively elevated from controls to PC and was highest in mPDAC. SYPL-1 demonstrated excellent diagnostic performance, with an AUC of 0.965 for distinguishing rPDAC from healthy controls, and an AUC of 0.985 for distinguishing mPDAC from healthy controls. This study demonstrates the potential of SYPL-1 as an effective diagnostic marker for PC. SYPL-1 showed excellent diagnostic accuracy, especially in distinguishing rPDAC and mPDAC from healthy controls, outperforming traditional markers like CEA and CA19-9. Post surgery, the SYPL-1 levels significantly decreased, indicating its role in monitoring disease progression. CEA and CA19-9 also provided valuable diagnostic information, particularly for metastatic cancer. These findings suggest that SYPL-1 could be a promising tool for early diagnosis and monitoring of PC.

Serum SYPL1 is a promising biomarker for many cancers, including PC. Liu et al. [15] found that SYPL1 was upregulated in colon cancer tissue and that serum SYPL1 levels were significantly higher in patients with CRC than in patients with adenoma and intact patients (AUC: 0.94 sensitivity: 86%, specificity: 91%). They also found a statistically significant association with lymph node invasion and showed that the SYPL1 levels decreased in patients undergoing radical surgery for CRC. In this regard, SYPL1 is also detectable in circulation. Additionally, fecal SYPL1 can be reliably measured in stool samples, offering potential as a non-invasive biomarker. It could play a crucial role in population screening, early diagnosis, prognosis prediction, and monitoring the therapeutic effects of CRC [12]. In a study conducted in PTC tissue, the different expression levels of SYPL1 suggested that it may be valuable as a biomarker [14]. The ectopic expression of SYPL1 in HCC tissues predicted poor prognosis of HCC patients with reduced overall survival and disease-free survival rates [13]. Overall, SYPL1 shows significant promise in oncology, especially for early detection and monitoring of cancer progression. The fact that SYPL1 is present in both serum and fecal samples suggests its potential for non-invasive diagnostic applications, making it a valuable tool for early cancer detection and population screening. Additionally, the decline in the SYPL1 levels following surgical intervention indicates its utility in monitoring treatment efficacy and disease recurrence. Its association with poor prognosis in certain cancers further highlights its potential as a prognostic biomarker. Given these capabilities, SYPL1 could become a key marker in the management of cancer, aiding not only in diagnosis but also in predicting outcomes and guiding therapeutic strategies.

However, the mechanism through which SYPL1 promotes the initiation and progression of tumors and the role of SYPL1 in PDAC remain unclear. Song et al. [11] found that high SYPL1 levels in 76 PDAC tumor tissues indicated poor prognosis and SYPL1 supported cell proliferation both in vivo and in vitro. This suggests that SYPL1 may play a crucial role in the aggressive nature of PDAC by promoting tumor growth and proliferation. The fact that elevated SYPL1 levels are linked to worse outcomes highlights its potential as a prognostic marker, indicating that higher SYPL1 expression may be a sign of more advanced or aggressive disease. Additionally, the ability of SYPL1 to support cell proliferation suggests that it could be involved in mechanisms that drive tumor progression, making it a potential target for future therapeutic strategies. These findings reinforce the importance of SYPL1 not only as a diagnostic biomarker but also as a molecule involved in the pathophysiology of PDAC. In the present study, serum SYPL-1 levels showed excellent diagnostic accuracy in distinguishing rPDAC patients from healthy individuals. Separately, when comparing resectable rPDAC with mPDAC, SYPL-1 also demonstrated moderate diagnostic utility, suggesting its potential role not only in early detection but also in monitoring disease progression.

In patients who underwent surgical resection, the SYPL-1 levels showed a significant decrease at 6 months postoperatively. In distinguishing pancreatic cancer from controls, SYPL-1 showed excellent diagnostic accuracy, with a sensitivity of 92.4% and specificity of 78.4% at the optimal cut-off value of 12. In differentiating metastatic from non-metastatic pancreatic cancer, SYPL-1 showed moderate diagnostic accuracy with 52.2% sensitivity and 100% specificity at a cut-off value of 19. These results showed that SYLP-1 can be used as a biomarker to differentiate rPDAC patients from mPDAC patients. Serum SYPL1 levels may be a potential biomarker for PDAC screening, early diagnosis, prognosis prediction and monitoring of therapeutic effects. At the same time, SYPL1 was found to have excellent diagnostic performance in distinguishing rPDAC and mPDAC from the controls. These results indicate that SYPL-1 could be a promising biomarker not only for PDAC screening and early diagnosis but also for predicting prognosis and monitoring treatment efficacy. Overall, SYPL-1 shows great promise in improving the clinical management of rPDAC and mPDAC, warranting further research to validate its utility in clinical practice.

In the current study, CEA demonstrated good diagnostic performance, with 89.2% sensitivity and 88.1% specificity at a cut-off value of 2.5. CA19-9 also showed excellent performance, with 82.1% sensitivity and 100% specificity at a cut-off value of 20. In differentiating metastatic from non-metastatic pancreatic cancer, SYPL-1 showed moderate diagnostic accuracy, with 52.2% sensitivity and 100% specificity at a cut-off value of 19. CA19-9 demonstrated fair performance, with 40.9% sensitivity and 100% specificity at both cut-off values of 3000 and 3500. According to the information given in Table 4, we reached 97.3% specificity with a cut-off of 3000 and 100% specificity with a cut-off of 3500. High-specificity tests are often used as confirmatory tests. In a high-specificity test, a positive result indicates the presence of disease with high accuracy. With these values, if a person’s result is above 3500, this indicates a definite mPDAC. The diagnostic value of SYLP is therefore important. SYPL1 offers greater sensitivity than CA19-9 and CEA while maintaining high specificity. This enhanced performance indicates its strong potential as a complementary or alternative biomarker for rPDAC, particularly for improving early detection and diagnostic accuracy.

These findings suggest that while SYPL-1, CEA, and CA19-9 can be valuable in differentiating rPDAC from controls and metastatic from non-metastatic disease, SYPL-1 shows considerable promise as a biomarker for early detection, disease monitoring, and prognosis prediction in rPDAC. Panel analysis of serum CEA, CA19-9 and SYLP1 may show a stronger diagnostic efficacy in rPDAC.

Inflammation plays a significant role in tumor progression, invasion, and metastatic spread. Nurmi et al. [21] suggest that both CRP and CA19-9 are valuable biomarkers in predicting the prognosis of PC. Notably, the novel prognostic score combining CRP and CA19-9 demonstrates strong utility as a preoperative tool for estimating survival outcomes. This score offers a simple yet effective method to assist in the clinical management of PC, enabling better risk stratification and aiding in decision-making for treatment plans. This innovative prognostic score, which combines CRP and CA19-9, proves to be an effective preoperative tool for predicting survival outcomes. Further validation of this prognostic score in larger, prospective studies would be beneficial for its implementation in clinical practice. Preoperative CRP was also an independent poor prognostic factor for overall survival and disease-free survival of patients with rPDAC [22]. In our study, inflammatory response, as measured by CRP, demonstrated a progressive increase from controls to rPDAC and was highest in mPDAC. CRP levels progressively increased from healthy controls to rPDAC and were highest in mPDAC, indicating that inflammation intensifies with disease progression. These findings highlight the role of systemic inflammation in pancreatic cancer and support CRP as a potential prognostic inflammatory biomarker. These findings support the use of CRP and CA19-9 as key biomarkers for prediction in PC, and further validation in larger, prospective studies will be essential for their clinical implementation.

The measurement of serum SYPL1 in PC and mPDAC is a strength of our study, as it is the first study to explore this marker in this context. One of the strengths of our study lies in the careful selection of patients, categorizing them into resectable, mPDAC, and postoperative 6-month groups. However, there are several limitations to consider. The relatively small sample size limits the generalizability of the findings. Additionally, the reduction in the number of patients in the postoperative group, from 39 to 20, due to factors such as patient relocation or mortality, should be considered when interpreting the results. These factors may have introduced selection bias and could impact the robustness of this study’s conclusions. Further research with larger sample sizes and more diverse patient cohorts would be beneficial to validate these findings.

Serum SYPL1 levels may be a potential biomarker for rPDAC screening, early diagnosis, prognosis prediction and monitoring of therapeutic effects. At the same time, SYPL1 was found to have excellent diagnostic performance in distinguishing rPDAC and mPDAC from healthy controls. Based on our findings, the significant postoperative decrease in SYPL-1 levels—like the behavior of CA19-9—suggests that SYPL-1 may indeed be useful for postoperative monitoring and assessing treatment response in PDAC patients. Furthermore, when examining the correlation analyses, SYPL-1 appears to exhibit a distinct pattern compared to traditional tumor markers, such as CA19-9 and CEA. Notably, SYPL-1 showed a negative correlation with CEA in the mPDAC group, suggesting it may be associated with a different biological pathway or mechanism involved in tumor behavior or progression. Given these observations, we believe that SYPL-1 could offer complementary clinical value alongside existing biomarkers and may provide insights into alternative molecular processes in pancreatic cancer, warranting further mechanistic investigations. Panel analyses of serum CEA, CA19-9 and SYLP1 may show a stronger diagnostic efficacy in rPDAC. Our study demonstrated that serum SYPL1 levels significantly decreased 6 months after surgical resection in rPDAC patients, suggesting that SYPL1 may be useful in monitoring treatment response and disease progression. This pattern is similar to that of established markers like CA19-9. Therefore, SYPL1 shows promise as a non-invasive biomarker for tracking the clinical course of pancreatic cancer and evaluating treatment effectiveness. However, further prospective studies with larger cohorts are needed to validate its utility in routine clinical follow-up.

As highlighted by Cocca et al. [23], the early diagnosis of pancreatic cancer remains one of the greatest challenges due to the lack of specific symptoms and the limited sensitivity of available biomarkers such as CA19-9. Beyond CA19-9, advanced imaging techniques (e.g., EUS, MRI, and contrast-enhanced CT) and novel biomarker approaches, including liquid biopsies and multi-omics strategies, are being explored to improve early detection. Our study contributes to this ongoing effort by investigating SYPL-1 as a potential serum biomarker that may enhance diagnostic accuracy, particularly in combination with existing tools.

However, more mechanistic studies are needed to fully elucidate SYPL-1’s oncogenic roles. SYPL-1 contributes to PDAC progression by inhibiting apoptosis through the suppression of ROS-induced ERK activation, thereby promoting tumor cell survival. This suggests that SYPL-1 plays a role in oncogenesis by modulating oxidative stress and key survival signaling pathways. While the mechanisms may vary across tissue types, its anti-apoptotic effect appears to be a common oncogenic feature [11]. While the precise molecular mechanisms of SYPL-1 in cancer remain under investigation, recent studies suggest that SYPL-1 may contribute to tumorigenesis by regulating cell proliferation, vesicle trafficking, and membrane dynamics. In PDAC, SYPL-1 overexpression has been associated with enhanced tumor cell growth and poor prognosis [11]. SYPL1, which belongs to the SYP family, was originally regarded as a neuroendocrine-related protein. Additionally, its role in other cancers such as colorectal, HCC, and thyroid carcinoma suggests a potential function in tumor progression and metastasis through distinct, tissue-specific pathways [11].

SYPL1 is a component of transport vesicles and is expressed in both neuronal and non-neuronal tissues [11]. Its localization within membrane compartments enables it to participate in intracellular trafficking and signaling pathways relevant to tumor biology. Notably, SYPL1 has been implicated in regulating the NF-κB signaling pathway, which plays a central role in inflammation, tumor proliferation, and progression [24]. Additionally, SYPL1 is associated with epithelial–mesenchymal transition (EMT) and has shown functional relevance in PDAC, CRC, HCC, and PTC [11,12,13,14,15,16]. SYPL1 may play a vital role in the incidence and development of PTC, serving as a potential biomarker for the diagnosis of PTC [14]. Serum SYPL1 might be an outstanding marker for CRC diagnosis, especially for patients with low CEA levels [15], and fecal SYPL1 might be a potential biomarker for CRC screening, early diagnosis, prediction and therapeutic effect monitoring [12]. As shown by Zhang et al. [17], SYPL1 contributes to CRC progression through the circ_0004104/miR-493-5p/SYPL1 regulatory axis. Elevated SYPL1 expression is associated with poor prognosis, while its downregulation—either directly or via circ_0004104 knockdown—suppresses tumor growth, suggesting SYPL1 may serve as a potential marker for cancer progression and treatment response in CRC. In HCC, SYPL1 overexpression is associated with poor prognosis and may promote tumor proliferation and invasion through EMT [13]. The SYPL1 level can be used with high specificity in diagnosing BC [16]. Overall, SYPL1 appears to influence cancer development through anti-apoptotic signaling, inflammation, EMT regulation, and intracellular trafficking, though further studies are needed to fully define its tumor-specific roles. These findings support SYPL1 as a valuable biomarker for early detection, prognostic evaluation, and disease monitoring in multiple malignancies.

The desmoplastic stroma is known to contribute to therapy resistance and tumor progression in PDAC by creating a physical barrier to drug delivery and promoting a pro-tumorigenic microenvironment. However, our study did not specifically investigate stromal components or their role in treatment resistance, as our focus was on evaluating SYPL-1 as a serum biomarker. As noted by Ramesh et al. [25], the dense desmoplastic stroma in PDAC not only promotes tumor progression but also creates a major barrier to effective drug delivery and immune cell infiltration.

Prospective studies, with a longer follow-up and including more patients, are needed to confirm our results. Overall, the findings suggest that combining SYPL-1, CEA, and CA19-9 could improve diagnostic accuracy and clinical decision-making in PC, offering valuable insights for early detection and monitoring of metastatic progression. Further validation in larger cohorts is warranted to confirm these findings and refine the use of these biomarkers in clinical practice. The future of serum markers in rPDAC likely lies in the combination of multiple markers, including SYPL-1, to improve diagnostic accuracy, early detection, and monitoring of therapeutic efficacy. Research into novel biomarkers and their molecular mechanisms continues to be a critical area of focus in the fight against rPDAC. Further research is needed to elucidate the precise molecular mechanisms underlying SYPL1’s involvement in cancer progression and its potential therapeutic applications.

## Figures and Tables

**Table 1:** Comparison of tumor markers and laboratory parameters among the control, resectable pancreatic ductal adenocarcinoma (rPDAC), and metastatic (mPDAC) groups.

	Control (*n* = 67)	rPDAC (*n* = 39)	mPDAC (*n* = 22)	
	Mean ± SD or Median (25p–75p)	Mean ± SD or Median (25p–75p)	Mean ± SD or Median (25p–75p)	*p*
Age (Year)	44.8 ± 13.84 ^a^	64.57 ± 9.67 ^b^	63.22 ± 9.44 ^b^	<0.001 †
Tumor diameter (mm)	-	3.2 (2.8–4.5)	4 (2.5–4.5)	0.645 ¶
Metastatic lymph node number	-	3 (0–6)	-	-
SYPL-1	7.43 ± 3.32 ^a^	15.89 ± 2.00 ^b^	20.01 ± 4.03 ^c^	<0.001 *
CEA	1.34 (1.06–2) ^a^	4.86 (2.58–9.4) ^b^	7.74 (3.66–25.6) ^b^	<0.001 *
CA19-9	6.89 (4.42–10.6) ^a^	330.25 (71.22–967.6) ^b^	1302.5 (251–9314) ^b^	<0.001 *
AFP	3.06 (2.19–3.97)	2.86 (1.83–4.54)	2.21 (1.71–2.99)	0.116 *
WBC	7.3 (6.3–8.4)	7.65 (6.2–8.3)	6.3 (5.8–8.6)	0.642 *
HCT	39.05 ± 4.82 ^a^	35.09 ± 4.27 ^b^	33.73 ± 4.98 ^b^	<0.001 †
PLT	274,712 ± 62,638	263,763 ± 90,355	234,865 ± 107,259	0.130 †
FBG	89 (73–98) ^a^	126.5 (105–176) ^b^	127.5 (94–163) ^b^	<0.001 *
Urea	26.58 ± 8.63 ^a^	32.92 ± 15.94 ^b^	29.3 ± 10.58 ^b^	0.030 †
Creatinine	0.71 ± 0.17 ^a^	0.82 ± 0.29 ^b^	0.68 ± 0.19 ^b^	0.020 †
AST	17 (15–24) ^a^	41 (16–104) ^b^	25 (19–55) ^b^	<0.001 *
ALT	18.5 (11–27) ^a^	44 (14–136) ^b^	28 (16–48) ^b^	0.001 *
Albumin	4.63 (4.45–4.86) ^a^	4.1 (3.55–4.33) ^b^	3.37 (3.03–4) ^b^	<0.001 *
CRP	2.31 (0.88–5.61) ^a^	6.5 (2.6–15.8) ^b^	22.25 (8.5–57.8) ^c^	<0.001 *
Tot. Bilirubin	0.37 (0.24–0.51) ^a^	1.3 (0.5–3.79) ^b^	1.2 (0.43–2.84) ^b^	<0.001 *
D. Bilirubin	0.16 (0.12–0.22) ^a^	1.17 (0.2–2.69) ^b^	0.5 (0.18–2.12) ^b^	<0.001 *
Amylase	55 (45–70)	59 (38.5–89.5)	57 (38–65)	0.410 *
ALP	71 (55–85) ^a^	210 (108–420) ^b^	155 (116–335) ^b^	<0.001 *
GGT	18.5 (11.5–24.5) ^a^	201 (30–735) ^b^	139 (68–390) ^b^	<0.001 *

†: one-way ANOVA; *: Kruskal–Wallis test; ¶: Mann–Whitney U test was applied. ^a,b^: Different superscript letters indicate differences between groups.

**Table 2:** Pre-operative and post-operative comparison of laboratory parameters in resectable pancreatic ductal adenocarcinoma (rPDAC) patients.

	Preoperative (*n* = 39)	Postoperative (*n* = 20)	
	Mean ± SD or Median (25p–75p)	Mean ± SD or Median (25p–75p)	*p* Value
SYPL-1 (ng/mL)	15.82 ± 1.93	11.26 ± 2.22	<0.001 †
CEA (ng/mL)	5.17 (2.4–9.59)	7.7 (3.1–9.55)	0.173 *
CA19-9 (ng/mL)	330.13 (170–950.67)	55 (44.55–78)	<0.001 *
AFP (ng/mL)	2.55 (1.77–3.4)	2.32 (1.9–3.8)	0.841 *
WBC (10^3^/µL)	7.73 (6.96–8.65)	8.1 (7.25–9.05)	0.550 *
HCT (%)	34.94 ± 3.79	35.09 ± 3.57	0.898 †
PLT (10^6^/µL)	270.60 ± 53.31	304.80 ± 175.31	0.409 †
FBG (mg/dL)	130 (107.5–188)	148.5 (110.5–169)	0.526 *
Urea (mg/dL)	32.3 ± 17.53	31.1 ± 13.84	0.823 †
Creatinine (mg/dL)	0.82 ± 0.25	0.78 ± 0.29	0.562 †
AST (U/L)	55.5 (22.5–142.5)	25 (19.5–38.5)	0.028 *
ALT (U/L)	110.5 (21–255)	22 (17–36)	0.019 *
Albumin (g/dL)	4.03 (3.5–4.32)	3.22 (2.89–3.58)	0.004 *
CRP (mg/L)	5.23 (2.92–17)	5.45 (3.53–11.1)	0.970 *
T. Bilirubin (mg/dL)	1.9 (0.6–6.53)	0.9 (0.75–1.25)	0.011 *
D. Bilirubin (mg/dL)	1.2 (0.25–5.28)	0.65 (0.42–0.88)	0.013 *
Amylase (U/L)	66.5 (39.5–95)	55.5 (46–70)	0.148 *
ALP (U/L)	364 (115.5–550)	102.5 (81–116)	0.002 *
GGT (U/L)	591.5 (109–949.5)	40 (32–52)	<0.001 *

†: Paired *t* test; *: Wilcoxon test was applied.

**Table 3:** Correlation analysis of SYPL-1, tumor markers, and laboratory parameters in groups.

		Resectable Pancreatic Ductal Adenocarcinoma (rPDAC) (*n* = 39)					Metastatic PDAC (*n* = 22)			
Variables	r/*p* Value	SYPL-1	CEA	CA19-9	Tumor Diameter	Lymph Node Number	SYPL-1	CEA	CA19-9	Tumor Diameter
SYPL-1	r		−0.094	−0.252	−0.141	−0.084		−0.530	−0.407	−0.196
	*p*		0.580	0.133	0.392	0.612		0.009	0.060	0.370
CEA	r			0.550	0.131	0.004			0.623	0.364
	*p*			<0.001	0.439	0.980			0.002	0.087

Spearman’s correlation analysis was applied.

**Table 4:** ROC analysis results for diagnostic efficacy of SYPL-1, CEA, CA19-9.

	Variable	AUC	95% CI	*p* Value	Cut-Off	Sensitivity	Specificity
**C vs. rPDAC**	SYPL-1	0.965	0.928–1	<0.001	12	100%	92.4%
	CEA	0.857	0.773–0.941	<0.001	2.5	78,4%	82.1%
	CA19-9	0.924	0.857–0.991	<0.001	20	89.2%	88.1%
					60	75.7%	100%
**C vs. mPDAC**	SYPL-1	0.985	0.964–1	<0.001	12	100%	92.4%
					14	100%	95.5%
	CEA	0.965	0.921–1	<0.001	2.5	95.7%	82.1%
					3	91.3%	91.0%
	CA19-9	0.986	0.963–1	<0.001	20	95.5%	88.1%
					60	90.9%	100%
**rPDAC vs. mPDAC**	SYPL-1	0.771	0.641–0.900	0.001	19	52.2%	97.4%
					20	47.8%	100%
	CEA	0.647	0.505–0.790	0.060	-	-	-
	CA19-9	0.701	0.553–0.850	0.010	3000	40.9%	97.3%
					3500	40.9%	100%

## Data Availability

The datasets used and/or analyzed during the current study are available from the corresponding author on reasonable request.
